# Mood States and Everyday Creativity: Employing an Experience Sampling Method and a Day Reconstruction Method

**DOI:** 10.3389/fpsyg.2019.01698

**Published:** 2019-07-19

**Authors:** Wei Han, Xue Feng, Mi Zhang, Kaiping Peng, Dan Zhang

**Affiliations:** ^1^Department of Psychology, Tsinghua University, Beijing, China; ^2^Department of Psychology, Yangtze University, Jingzhou, China

**Keywords:** creativity, mood state, experience sampling method, day reconstruction method, daily life

## Abstract

Investigating the mood-creativity relationship in everyday life is important for innovation promotion in organizational management. This study explores the relationship between mood states and everyday creativity using two different measurement methods. Both the experience sampling method (ESM) and the day reconstruction method (DRM) were simultaneously applied to conduct a 15-day follow-up study of the relationship between 10 typical positive and negative moods and creativity in daily situations. In total, 31 corporate employees participated in the study. Participants reported their mood states and creativity at three time points per day by using ESM; they also recalled and reported their mood states and creativity for at least three major events per day at the end of each day by using DRM. In total, we collected 935 valid measurements from ESM and 1260 valid measurements from DRM. The results revealed that highly active, positive moods including happiness, concentration, feeling active and interested had significant positive correlations with creativity, while the low-activity, negative mood states of feeling tired and sleepy were associated with low creativity. Both DRM-based and ESM-based results were largely consistent in measuring individual’s mood states and everyday creativity. To our knowledge, this is the first study to conduct DRM for mood-creativity relationship. The high consistency between the two daily research methods provides further empirical evidence toward a comprehensive understanding of mood and creativity. As DRM imposes a lessened respondent load on the participants as compared to ESM, our results suggest DRM as a promising tool for further mood-creativity research.

## Introduction

Creativity, the ability to engage in both original and practical production, has been described as the most important economic resource of the 21st century (Sternberg and Lubart, 1999). In fact, whether in terms of the historical contribution that creativity has made to the development of human society, or its ability to adapt to contemporary society’s political, economic, and cultural development and to solve practical problems, creativity has long been of extraordinary importance to human society (Kaufman and Sternberg, 2010). Traditionally, creativity has been regarded as a relatively stable individual trait (e.g., Gough, 1979; Tierney and Farmer, 2002; Zhou and Shalley, 2003). More recently, however, scholars have begun to consider creativity as an unstable state that can change according to individual and situational factors (Shalley and Gilson, 2004; George, 2007). In particular, mood, as a state characteristic that is sensitive to transient situational factors, has attracted the attention of many researchers (Isen, 1999; George and Zhou, 2002; Baas et al., 2008; Davis, 2009).

The relationship between mood state and creativity has been extensively investigated in laboratory studies. Recent meta-analyses have concluded that an induced positive mood often tends to facilitate creative performance compared to neutral mood (e.g., Hirt et al., 1997; De Dreu et al., 2008). Correspondingly, it has been suggested that positive moods are associated with looser information processing, therefore facilitating divergent thinking, and novelty-seeking behaviors (Baas et al., 2008). The findings on negative moods, however, remains controversial. While some studies showed that negative moods promote creative performance compared to neutral moods (e.g., Adaman and Blaney, 1995; Carlsson et al., 2000; Clapham, 2001), others reported a negative or non-significant effect (e.g., Mikulincer et al., 1990; Vosburg, 1998; Göritz and Moser, 2003; Verhaeghen et al., 2005). It has been postulated that negative moods might contribute to creativity in a complex manner, as proposed by the dual-tuning model, the affective shift model, etc. (George and Zhou, 2007; Bledow et al., 2013).

Measuring moods and creativity in daily situations is of value when developing a better understanding of how creativity as a state is affected by temporary situational factors. To date, the daily studies on the mood-creativity relationship have been conducted mainly using the experience sampling method (ESM). The ESM, (i.e., repeated sampling of experiences in real time in natural contexts), is believed to overcome the problem of recall bias and intuition errors that can occur in traditional retrospective questionnaire surveys (Shiffman et al., 2008). In the context of the mood-creativity relationship, To et al. (2012) examined the effect of mood valence and activation level on the creative process engagement of individuals on a daily basis, reporting that the moods with high activation characteristics were positively associated with creative process engagement, regardless of their valence level. Silvia et al. (2014) employed the ESM to investigate mood-creativity relationship and found that people were more likely to be creative when they were happy than when they were angry or sad. To date, it has been generally agreed that activating and positive mood states in daily life are associated with creative thoughts and activities (Baas et al., 2008; De Dreu et al., 2008; To et al., 2012; Conner and Silvia, 2015).

In contrast to the popularity of ESM in creativity studies, another type of important daily research method, the day reconstruction method (DRM), has not been applied to measure the mood-creativity relationship to date. DRM usually takes places at the end of the day. This method requires the participants to recall a sequence of behavioral episodes they have experienced during the day, noting their duration, location, social interaction, and activity. Participants are then asked to report their mood states for each episode (Morrison et al., 1999; Kahneman et al., 2004). As the measurement is *post hoc*, DRM data involves obtaining specific information for each behavior, while imposing a lessened respondent load on the participants as compared to ESM (Kahneman et al., 2004). Nevertheless, DRM has been suggested to be robust against recall bias, with consistent results as their ESM counterpart on affective ratings (Kahneman et al., 2004; Dockray et al., 2010; Kim et al., 2013). However, a systematic comparison between these two methods in the field of creativity is lacking. The daily study of creativity and its correlation with mood states could be substantially different from existing studies. Daily creativity is a complex concept distinct from basic mood states for one thing and, for another, the mood-creativity relationship is a second-order measurement that have not been investigated using DRM, let alone its consistency with ESM.

In the present study, a comparison between the two daily measurement methods of ESM and DRM was conducted in the context of the mood-creativity relationship. Both ESM and DRM were simultaneously applied to obtain self-reported mood states and creativity from a group of corporate employees for 15 days. Ten mood-state items were employed to describe the daily mood experiences at a fine-grained level. Compared to previous DRM studies, the present study had an extended number of recording days [2 days and 3 days, in Dockray et al. (2010) and Kim et al. (2013), respectively] and a comprehensive coverage of the mood state types, in order to conduct a more complete comparison between the two methods. While a possible difference between the results from the two methods may reveal possible limitations for one of the methods and provide further clues to the understanding of the mechanisms on mood-creativity relationships, stronger conclusions can be made if the two methods yield consistent findings.

## Materials and Methods

### Participants

Participants in this study comprised 31 corporate employees (8 female, average age 30.7 ± 6.4 years) from corporate Research and Development departments in Beijing and Shanghai. This study was carried out in accordance with the Declaration of Helsinki. The protocol was approved by the local Ethics Committee of the Department of Psychology, Tsinghua University. All participants provided written informed consent.

Given the specificity of the research methods, the participants were required to have stable working environments and few business trips during the data collection period. After the study, each participant received 50 RMB (approx. 8 US Dollars) per day as compensation for volunteering. Participants were provided with an analysis report of their creativity-mood relationship by the end of their participation.

### Procedure

All the participants received both ESM and DRM measurement. Before the study, the experimenter explained the procedure to the participants and asked them to sign the informed consent form. Assessment reports of participants’ creativity and moods in daily life were collected through the Psychorus research platform (HuiXin, China). To implement this, the participants were asked to install the Psychorus App on their mobile phones, and to complete the daily ESM and DRM questionnaires provided through this program. Psychorus sent four questionnaire notifications to the participants’ phones daily. Of these notifications, three were with regard to ESM questionnaires and one was with regard to DRM questionnaire; these notifications involved sound and vibration alerts. The volunteers were instructed to answer the questionnaires as soon as possible after each notification. All reported data were automatically uploaded to the server. The total duration of the study was 15 days.

ESM data collection: The participants received three ESM-questionnaire notifications every day, each at a random time point between 10:30 and 11:30, 13:30 and 14:30, and 15:30 and 16:30, respectively. After receiving the notification, each volunteer was required to complete a questionnaire on their mobile phone, reporting their activities as well as their relevant creativity and mood status during the past 30 min. Each push notification was continuously displayed in the notification bar of the mobile phone until the participant clicked on it and submitted the questionnaire. In cases where participants did not immediately begin the ESM questionnaire after receiving the notification, the system would remind them every 5 min until 1 h had passed, after which the push notification would disappear and the questionnaire would be closed.

DRM data collection: the participants received a DRM questionnaire notification at 21:00 every night, and were sent reminders to complete the DRM questionnaire from the time they received the first notification until 8:00 the following day. Following the DRM design proposed by Kahneman et al. (2004), the participants were asked to recall their day as a continuous series of behavioral episodes in a temporal order. While the original version of DRM required to report all relevant events, here, we required participants to report at least three major events per day, in order to reduce the burden of performing DRM for 15 days. The participants were explicitly informed that three major events were expected to be their most impressive events of the day but not necessarily the most creative ones. The participants were asked to name the events, determine the starting and ending time, and to describe the event content, location, and communication objects. Finally, the daily statuses of creativity and mood in each event period were evaluated, for which the questions used were the same as those used in the ESM evaluation.

### Measures

Creativity: Creativity was measured using a single item that we developed based on the common definitions of creativity, which is similar to previous daily studies (Silvia et al., 2014; Conner and Silvia, 2015). Specifically, our question was: “Overall, how creative were you over the past 30 min?” Participants were asked to rate their creativity using a moving bar on a horizontal scale (where leftmost = *not at all*, and rightmost = *very much*). Self-report score was then obtained according to the bar position, with 0 representing leftmost and 100 representing rightmost.

Mood states: Mood states were measured using a 10-item questionnaire similar to the Positive and Negative Affect Scale (PANAS; Muaremi et al., 2013). The selection of the 10 items followed a previous ESM study on affective ratings (Muaremi et al., 2013). This scale required the participants to self-report their mood states by scoring 10 items between 0 and 100 points (0 points for complete non-conformity, 100 points for excellent conformity, in the same way as for creativity). Specifically, the 10 items concerned the moods of relaxed, happy, concentrated, interested, active, tired, stressed, sleepy, angry, and depressed (five positive items and five negative items).

### Statistical Analysis

Correlations between the 10 mood states and creativity were calculated using both DRM and ESM methods, to provide a detailed comparison between the two methods with a fine-grained description from the mood perspective. The DRM data contained more activities conducted by the participants during the day, while the ESM data were only sampled at three random time points during the day. Therefore, the reported events of DRM data were greater in number than the ESM data. Consequently, the data analysis in this study consisted of two parts. The first part involved comparing the DRM data as a whole with the results from the ESM data as a whole (analysis of all data), while the second part concerned using the DRM and ESM scores provided by the same participants for the events with overlapping time periods to compare the results between the two methods at a single measurement level.

#### Analysis of All Data

First, all of the DRM and ESM data were analyzed as follows:

(1) The correlation between creativity and mood was explored on a single measurement level by pooling together the data across participants, ignoring the nested structure (i.e., stacked data). As a similar number of samples were acquired for each participant, similar results from (1) and (2) would imply that a consistent mood-creativity relationship at both levels, providing further evidence for understanding the underlying mechanism.
(2) To address the nested structure of the obtained daily data (i.e., each participant with multiple reports), the mood-creativity correlation coefficient was first calculated within each participant for both DRM and ESM data and statistical analyses were then performed at the across-participant level. Note that we did not employ the conventional multi-level analysis due to the relatively limited sample size and the aim to include all the 10 mood states.
(3) With mood states as independent variables and creativity scores as dependent variable, a multiple regression analysis was employed to explore the overall relationship between mood and creativity. Considering the sample size issue and based on the consistent results from (1) and (2) (see section “Results”), stacked data were used for regression. The regression analysis is expected to provide information about the explanatory power of the 10 mood states on creativity.
(4) Finally, the ESM and DRM results obtained in the above three analyses were compared, in which the correlations and differences between the two methods were explored via correlational analysis and difference tests.

#### Analysis of Matched Data

Using the ESM and DRM data that were matched with each other in terms of having the same participants and the event with overlapping time periods, we examined the correlation between the creativity and mood reported through each method. Events were considered to be matched if the time displayed in an ESM record (always a 30-min period) showed greater than 50% overlap within the time range of a DRM record. Next, employing correlation analysis and a paired sample *t*-test, we examined the correlations and differences between the creativity scores as well as between the mood scores obtained through the two methods.

## Results

### Recording Profiles

For the ESM data, 4 participants who answered less than 10 questionnaires were excluded from further analysis, meaning a total of 27 participants remained in the study. This corresponded to a total of 935 measurements of data; an average of 35 measurements per participant, 2.3 per participant per day, and a mean response rate of 77.8%. For the DRM data, 3 participants who reported less than 10 events in total were excluded from further analysis. This left a total of 28 participants remaining in the study. These participants reported 1, 260 events in total; an average of 45 events per participant, three per participant per day, and 65 min per reported event. Twenty-three out of the participants completed both the ESM and DRM recordings, therefore used in the following analysis.

### Mood-Creativity Correlations in DRM and ESM Recordings

The pairwise correlations between the 10 mood states were calculated using DRM and ESM records respectively (Tables 1, 2). In general, positive correlations were observed within the positive and the negative mood states, whereas negative correlations were obtained between pairs with one positive and one negative mood states. The correlation coefficients from the stacked and unstacked (nested) data were of comparable magnitudes, suggesting similar intra-mood relationships at the within- and across-participant levels. Similar correlation patterns were seen from both DRM and ESM data. While the largest correlation coefficients were obtained between angry and depression (ranging from 0.825 to 0.976, depending on the conditions), most of the pairwise correlations showed moderate magnitudes.

**TABLE 1:** Correlations between the 10 mood states (ESM).

	**Relaxed**	**Tired**	**Happy**	**Stressed**	**Concentrated**	**Sleepy**	**Active**	**Angry**	**Depressed**	**Interested**
Relaxed	1	–0.481^∗∗∗^	0.556^∗∗∗^	–0.409^∗∗∗^	0.179^∗∗∗^	–0.317^∗∗∗^	0.170^∗∗∗^	–0.339^∗∗∗^	–0.343^∗∗∗^	0.354
Tired	–0.631^∗∗∗^	1	–0.427^∗∗∗^	0.363^∗∗∗^	–0.275^∗∗∗^	0.730^∗∗∗^	–0.211^∗∗∗^	0.429^∗∗∗^	0.443^∗∗∗^	–0.334
Happy	0.751^∗∗∗^	−0.414^*^	1	–0.281^∗∗∗^	0.398^∗∗∗^	–0.338^∗∗∗^	0.489^∗∗∗^	–0.225^∗∗∗^	–0.248^∗∗∗^	0.561
Stressed	−0.408^*^	0.424^*^	–0.203	1	0.142^∗∗∗^	0.304^∗∗∗^	0.090^∗∗∗^	0.252^∗∗∗^	0.278^∗∗∗^	–0.086
Concentrated	0.324	–0.175	0.483^∗∗^	0.286	1	–0.324^∗∗∗^	0.502^∗∗∗^	–0.163^∗∗∗^	–0.197^∗∗∗^	0.524
Sleepy	–0.620^∗∗∗^	0.953^∗∗∗^	−0.385^*^	0.439^*^	–0.190	1	–0.254^∗∗∗^	0.408^∗∗∗^	0.443^∗∗∗^	–0.335
Active	0.504^∗∗^	–0.211	0.728^∗∗∗^	0.120	0.578^∗∗∗^	–0.222	1	–0.060	–0.052	0.525
Angry	–0.308	0.618^∗∗∗^	–0.241	0.301	–0.200	0.612^∗∗∗^	–0.078	1	0.843^∗∗∗^	–0.265
Depressed	–0.290	0.623^∗∗∗^	–0.269	0.346^*^	–0.280	0.612^∗∗∗^	–0.068	0.965^∗∗∗^	1	–0.270
Interested	0.584^∗∗∗^	–0.320	0.769^∗∗∗^	–0.017	0.527^∗∗^	–0.309	0.708^∗∗∗^	–0.331	–0.317	1

^*^p < 0.05, ^∗∗^*p* < 0.01, ^∗∗∗^*p* < 0.001. Correlation coefficients using unstacked (nested) and stacked data are shown below and above the diagonal, respectively. The unstacked correlation coefficients were the averaged coefficients of the within-participant correlations.

**TABLE 2:** Correlations between the 10 mood states (DRM).

	**Relaxed**	**Tired**	**Happy**	**Stressed**	**Concentrated**	**Sleepy**	**Active**	**Angry**	**Depressed**	**Interested**
Relaxed	1	–0.413^∗∗∗^	0.656^∗∗∗^	–0.543^∗∗∗^	0.245^∗∗∗^	–0.333^∗∗∗^	0.325^∗∗∗^	–0.314^∗∗∗^	–0.354^∗∗∗^	0.470^∗∗∗^
Tired	–0.332	1	–0.378^∗∗∗^	0.387^∗∗∗^	–0.303^∗∗∗^	0.745^∗∗∗^	–0.250^∗∗∗^	0.423^∗∗∗^	0.446^∗∗∗^	–0.333^∗∗∗^
Happy	0.763^∗∗∗^	–0.063	1	–0.333^∗∗∗^	0.444^∗∗∗^	–0.378^∗∗∗^	0.564^∗∗∗^	–0.303^∗∗∗^	–0.298^∗∗∗^	0.664^∗∗∗^
Stressed	–0.455^∗∗^	0.673^∗∗∗^	–0.170	1	0.026	0.373^∗∗∗^	–0.042	0.351^∗∗∗^	0.364^∗∗∗^	–0.160^∗∗∗^
Concentrated	0.672^∗∗∗^	–0.144	0.753^∗∗∗^	–0.080	1	–0.353^∗∗∗^	0.498^∗∗∗^	–0.168^∗∗∗^	–0.221^∗∗∗^	0.502^∗∗∗^
Sleepy	–0.296	0.941^∗∗∗^	–0.028	0.769^∗∗∗^	–0.053	1	–0.308^∗∗∗^	0.461^∗∗∗^	0.461^∗∗∗^	–0.345^∗∗∗^
Active	0.603^∗∗∗^	0.081	0.796^∗∗∗^	0.065	0.751^∗∗∗^	0.156	1	–0.091^∗∗∗^	–0.124^∗∗∗^	0.598^∗∗∗^
Angry	–0.199	0.545^∗∗^	–0.108	0.568^∗∗^	–0.157	0.642^∗∗∗^	0.121	1	0.823^∗∗∗^	–0.234^∗∗∗^
Depressed	–0.240	0.551^∗∗^	–0.120	0.584^∗∗∗^	–0.225	0.638^∗∗∗^	0.082	0.976^∗∗∗^	1	–0.247^∗∗∗^
Interested	0.686^∗∗∗^	–0.030	0.802^∗∗∗^	–0.067	0.688^∗∗∗^	–0.004	0.821^∗∗∗^	0.027	0.027	1

*^∗∗^*p* < 0.01, ^∗∗∗^*p* < 0.001. Correlation coefficients using unstacked (nested) and stacked data are shown below and above the diagonal, respectively. The unstacked correlation coefficients were the averaged coefficients of the within-participant correlations.*

**FIGURE 1:**
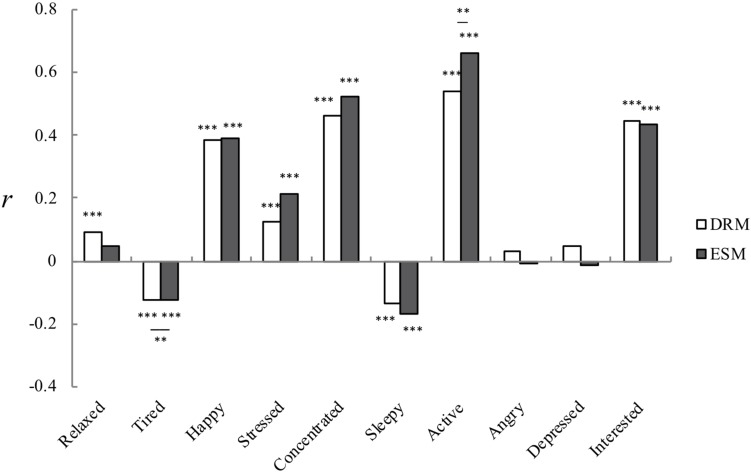
Correlation coefficients between daily creativity and mood states based on the stacked data (^∗∗^*p* < 0.01, ^∗∗∗^*p* < 0.001).

The correlation analyses based on the stacked data were performed using 1, 260 DRM records and 935 ESM records separately. As shown in Figure 1, the DRM results indicates that the moods of relaxed, happy, concentrated, active, interested and stressed were significantly and positively correlated with the scores for daily creativity (*r*_relaxed_ = 0.09, *p* < 0.001, *r*_happy_ = 0.39, *p* < 0.001, *r*_concentrated_ = 0.46, *p* < 0.001, *r*_active_ = 0.54, *p* < 0.001, *r*_interested_ = 0.45, *p* < 0.001, *r*_stressed_ = 0.12, *p* < 0.001); in contrast, the two negative moods of tired, sleepy were determined to be significantly and negatively correlated with creativity (*r*_tired_ = −0.12, *p* < 0.001; *r*_sleepy_ = −0.13, *p* < 0.001), while neither of the negative moods of angry and depressed were significantly correlated with daily creativity. The ESM data indicates that the moods of happy, concentrated, active, interested, and stressed had a significant positive correlation with daily creativity scores (*r*_happy_ = 0.39, *p* < 0.001, *r*_concentrated_ = 0.46, *p* < 0.001, *r*_active_ = 0.54, *p* < 0.001, *r*_interested_ = 0.45, *p* < 0.001, *r*_stressed_ = 0.21, *p* < 0.001), while the two negative moods of tired and sleepy were significantly and negatively correlated with creativity (*r*_tired_ = −0.12, *p* < 0.001, *r*_sleepy_ = −0.13, *p* < 0.001). The three moods of relaxed, angry, and depressed were not significantly correlated with creativity. Statistically significant differences in mood-creativity correlation strength were found for the two moods of stressed and active. More positive correlations were obtained using ESM for them, as compared to DRM (stressed: −0.215 vs. 0.122, *Z* = 2.211, *p* < 0.05; active: 0.659 vs. 0.537, *Z* = −4.419, *p* < 0.01).

**FIGURE 2:**
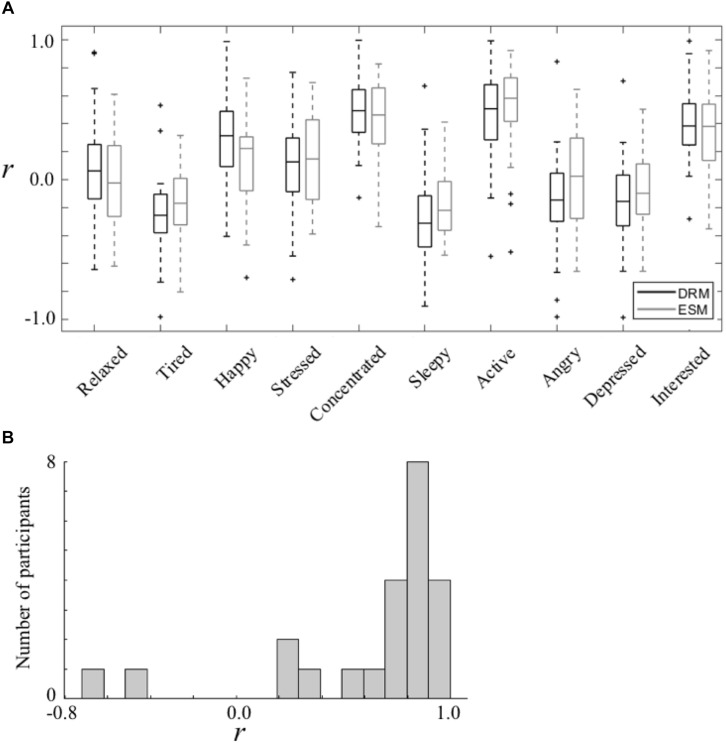
**(A)** Boxplots of the within-participant mood-creativity correlations. **(B)** Distribution of the participant-wise correlation between the 10-dimension mood-creativity constructs using ESM and DRM data.

The nested data structure was then taken into consideration by inspecting the averages of the within-participant correlations. Denoting the ESM-based coefficients based on ESM data as *r*_ESM_ and the DRM-based coefficients as *r*_DRM_, Figure 2A shows the distribution of such correlation coefficients from all participants. The directions of the correlations were similar to the results with stacked data (Figure 1). The differences between *r*_ESM_ and *r*_DRM_ were further examined by using paired sample *t*-tests. No significant differences were found between *r*_ESM_ and *r*_DRM_ (Table 3), indicating comparable mood-creativity correlation strengths for all the 10 mood states.

**TABLE 3:** Mood-creativity correlations with DRM and ESM data.

	***r*_DRM_**	***r*_ESM_**	**Difference**	***t*(22)**	***p***
Relaxed	0.08±0.37	−0.01±0.37	0.09±0.40	1.119	0.275
Tired	−0.23±0.29	−0.16±0.24	−0.07±0.33	–1.082	0.291
Happy	0.30±0.31	0.17±0.32	0.13±0.36	1.829	0.080
Stressed	0.09±0.36	0.11±0.33	−0.02±0.32	–0.318	0.754
Concentrated	0.48±0.27	0.45±0.30	0.03±0.29	0.449	0.658
Sleepy	−0.27±0.35	−0.22±0.24	−0.05±0.35	–0.748	0.462
Active	0.47±0.31	0.51±0.33	−0.04±0.23	–0.842	0.408
Angry	−0.15±0.38	−0.05±0.34	−0.10±0.39	–1.301	0.206
Depressed	−0.16±0.34	−0.08±0.34	−0.09±0.44	–0.948	0.353
Interested	0.38±0.29	0.32±0.31	0.06±0.40	0.755	0.458

A further comparison was made by taking the 10 correlation coefficients from either DRM or ESM data as a single 10-dimension construct and calculating the correlation between the DRM-based and the ESM-based constructs for each individual participant. A large correlation coefficient would imply a similar level of the relative magnitude of different mood-creativity links between the two methods, whereas the direct comparison of the single-dimension correlation values (i.e., *r*_ESM_ vs. *r*_DRM_ under a certain mood state) focuses on absolute magnitudes. The between-construct correlation values were larger than 0.5 for 18 out of the 23 participants (*M* = 0.64, *SD* = 0.43, Figure 2B), and the mean value was significantly larger than zero [*t*(22) = 7.066, *p* < 0.001], further arguing for largely consistent results from both methods.

With the creativity scores as dependent variable and the 10 mood states as independent variables using DRM and ESM data separately, both models show that the mood states could significantly explain the state creativity (51.5% and 40.5% for ESM and DRM respectively, Table 4). Both models received significant contributions from the mood states of relaxed, happy, stressed, concentrated, and active. Tired, depressed and interested were found to have significant regression weights for DRM only. It is worthwhile to note that the regression did not suffer from a multi-collinearity problem, as the variance inflation factor (VIF) values were <4 for all the variables in both models.

**TABLE 4:** Regression results for creativity by the 10 mood states.

	**ESM**		**DRM**	
**Mood variable**	**Standardized β**	***t***	**Standardized β**	***t***
Relaxed	–0.109	–3.558^∗∗∗^	–0.181	–5.462^∗∗∗^
Tired	–0.002	–0.052	–0.070	−2.041^*^
Happy	0.165	4.887^∗∗∗^	0.197	5.436^∗∗∗^
Stressed	0.145	5.227^∗∗∗^	0.073	2.549^*^
Concentrated	0.198	6.642^∗∗∗^	0.219	7.895^∗∗∗^
Sleepy	–0.005	–0.15	0.068	1.944
Active	0.457	15.116^∗∗∗^	0.311	10.422^∗∗∗^
Angry	0.057	1.329	–0.026	–0.662
Depressed	–0.02	–0.448	0.161	4.046^∗∗∗^
Interested	0.054	1.726	0.150	4.636^∗∗∗^
Overall regression	*F*(10, 924) = 100.031^∗∗∗^		*F*(10, 1249) = 86.567^∗∗∗^	
	*R*^2^ = 0.520		*R*^2^ = 0.409	
	Adjust *R*^2^ = 0.515		Adjust *R*^2^ = 0.405	

^*^p < 0.05, ^∗∗∗^*p* < 0.001.

### Results From Matched Samples

By selecting the events with greater than 50% overlap of the durations in both ESM and DRM records, a total of 200 records from 11 participants were obtained.

**FIGURE 3:**
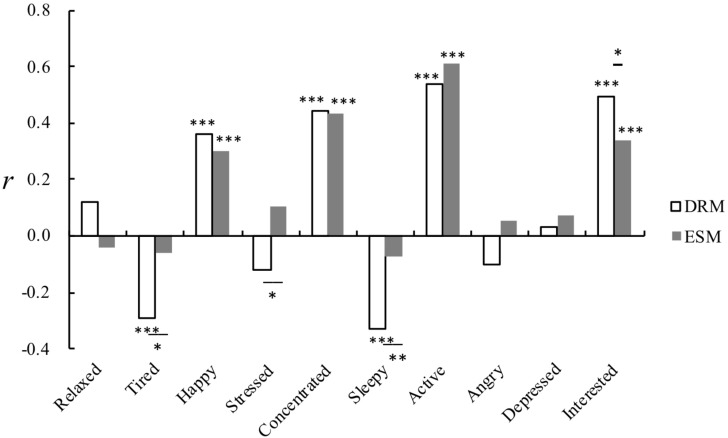
Creativity-mood correlations in the matched recordings (^*^p < 0.05, ^∗∗^*p* < 0.01, ^∗∗∗^*p* < 0.001).

As shown in Figure 3, the mood-creativity correlation analyses for the matched data collected via DRM show that the moods of happy, concentrated, active, and interested had a significant positive correlation with the daily creativity scores (*r*_happy_ = 0.36, *p* < 0.001, *r*_concentrated_ = 0.44, *p* < 0.001, *r*_active_ = 0.54, *p* < 0.001, *r*_interested_ = 0.50, *p* < 0.001); the moods of tired and sleepy had a significant negative correlation with creativity (*r*_tired_ = −0.29, *p* < 0.001, *r*_sleepy_ = −0.33, *p* < 0.001); the remaining four moods of relaxed, stressed, angry, and depressed were not significantly related to daily creativity. For ESM, the moods of happy, concentrated, active, and interested were found to be significantly and positively correlated with the daily creativity scores (*r*_happy_ = 0.30, *p* < 0.001, *r*_concentrated_ = 0.43, *p* < 0.001, *r*_active_ = 0.61, *p* < 0.001, *r*_interested_ = 0.34, *p* < 0.001); there was no significant correlation between the other moods and creativity. The findings on the four moods of happy, concentrated, active, and interested were consistent between ESM and DRM.

Notably, statistically significant differences in the mood-creativity correlation strength were found for the four moods of tired, stressed, sleepy, and interested. More negative correlations were obtained using DRM for tired and sleepy, as compared to ESM (tired: *r*_DRM_ = −0.293 vs. *r*_ESM_ = −0.058, *Z* = −2.382, *p* < 0.05; sleepy: *r*_DRM_ = −0.327 vs. *r*_ESM_ = −0.073, *Z* = −2.680, *p* < 0.01). A more positive correlation was observed using DRM for interested (*r*_DRM_ = 0.496 vs. *r*_ESM_ = 0.339, *Z* = 1.985, *p* < 0.05); and opposite correlation values were seen for stressed (*r*_DRM_ = −0.121 vs. *r*_ESM_ = 0.106, *Z* = −2.283, *p* < 0.05).

Finally, correlations and paired sample *t*-tests were performed to analyze the difference between DRM and ESM in terms of scale scores for matched events (Table 5). There were moderate positive correlations between the scores obtained by the two methods. While there was no significant difference in creativity scores between the two methods, the scores for the moods relaxed [*t*(199) = 2.096, *p* < 0.05], active [*t*(199) = 2.769, *p* < 0.01], and interested [*t*(199) = 2.383, *p* < 0.05] were significantly higher under DRM than under ESM. Nevertheless, these observed differences reached no less than 10% of the original scores, suggesting a small effect size. There was no significant difference between the two methods with regard to the other mood scores.

**TABLE 5:** Correlation and *t*-test results of the scale scores.

	**Correlation**	**Mean of DRM**	**Mean of ESM**	**Difference**	***t*(199)**	***p***
Creative	0.353^∗∗^	51.10±25.86	51.65±23.89	−0.55±28.34	0.277	0.782
Relaxed	0.435^∗∗^	70.89±24.02	66.97±25.74	3.92±26.49	−2.096^*^	0.037
Tired	0.522^∗∗^	34.57±26.99	32.22±24.10	2.35±25.11	–1.324	0.187
Happy	0.481^∗∗^	61.51±23.31	59.62±23.65	1.89±23.91	–1.118	0.265
Stressed	0.546^∗∗^	39.68±28.66	39.82±27.16	−0.13±26.62	0.072	0.943
Concentrated	0.321^∗∗^	66.01±22.55	65.69±21.16	0.33±25.49	–0.180	0.857
Sleepy	0.496^∗∗^	32.45±26.27	29.92±23.62	2.53±25.14	–1.423	0.156
Active	0.317^∗∗^	57.91±23.49	52.69±22.08	5.22±26.66	–2.769^∗∗^	0.006
Angry	0.542^∗∗^	20.35±22.55	20.71±22.76	−0.35±21.69	0.231	0.817
Depressed	0.634^∗∗^	18.41±19.72	20.02±21.10	−1.61±17.45	1.305	0.193
Interested	0.266^∗∗^	66.13±20.32	61.91±20.96	4.22±25.01	−2.383^*^	0.018

^*^p < 0.05, ^∗∗^*p* < 0.01.

## Discussion

In this study, we employed two kinds of common daily research methods (the ESM and the DRM) to measure individual creativity and mood states in daily situations. We investigated the associations between creativity and mood states in terms of the data obtained by the two different methods. The similarities and differences between two methods in measuring mood-creativity relationships were investigated. The results using the two methods were largely consistent, providing further evidence for the links between mood states and creativity in daily life situations.

Our results revealed a strong correlation between individuals’ moods and their reported creativity. For most of the 10 moods, the mood-creativity correlation was consistent between ESM and DRM. For example, the activating positive moods like happy, concentrated, active, and interested, as well as the activating negative mood of stressed, had significant positive correlations with creativity, while the deactivating negative moods of tired and sleepy were significantly and negatively correlated with creativity, indicating that a high-creativity state is usually accompanied by activating positive moods; a low-creativity state is often accompanied by deactivating negative moods. Moreover, instant mood states had strong predictive powers for creativity. Specifically, the findings show that the 10 mood states could account for 41–52% of creativity-score variation, with the positive moods as the significant contributors in both methods (Table 2). This aligns with conclusions in previous studies regarding mood-creativity correlations – mood states that are positive, activated, and promotion-focused are particularly likely to foster creative ideas (Schwarz and Clore, 2003; George and Zhou, 2007; Baas et al., 2008; De Dreu et al., 2008; To et al., 2012; Conner and Silvia, 2015).

At the level of individual participants, we compared the mood-creativity correlations between the two methods and found that for each participant the correlation showed consistent trends. Moreover, no significant difference was found in the mood-creativity correlation coefficients between the two methods, indicating that the two methods are consistent with each other in describing the overall situation of a single participant, and that DRM – like ESM – can provide an accurate and instant report of mood and creative intensity. This result is consistent with the findings in previous studies that compared DRM and ESM in terms of mood measurement (Kahneman et al., 2004; Dockray et al., 2010; Kim et al., 2013). However, a greater number of positive and negative moods were included and compared in this study than in previous studies. In addition, the comparison between the two method in terms of creativity shows that DRM can also serve as an effective tool for measuring the creativity of individuals in daily situations, in addition to measuring moods. Most importantly, the similarity of the mood-creativity correlations found by the two methods extend our previous understanding of DRM, showing its capability for studying second-order statistics such as the bivariate correlation.

Notably, we did observe some differences between the two methods when analyzing the matched data. The self-report scores of the positive mood states of relaxed, active, and interested were higher when using DRM than they were when using ESM. No differences were found for all the negative moods (Table 3). The mood-creativity correlations showed stronger correlation strengths obtained by DRM largely for the negative moods of tired, sleepy and stressed (but also stronger correlations for interested, see Figure 3) as compared to ESM. These differences suggest possibly distinct mechanisms for ESM and DRM, and further studies with better controlled scenarios are necessary to properly address these issues.

It should be worthwhile to mention that the participants were simultaneously measured by ESM and DRM on the same days. Such a practice is commonly adopted in recent studies (e.g., Dockray et al., 2010; Kim et al., 2013), as recording on the same days would help to control for cognitive factors that could differ substantially across days. Nevertheless, the acts of providing ESM reports could potentially enhance memory of the corresponding events, thus biasing the DRM reports at the end of the ESM day toward the ESM reports. Such a bias, however, is not likely to significantly undermine the effectiveness of our correlation-based comparison. First, the scale scores were only moderately correlated between the two methods (Table 5), in the range from ∼0.2 up to ∼0.6. Second, even if the participants could replicate the ESM-based scale scores to some degree in their DRM reports, as it would be difficult for them to “replicate” second-order statistics (i.e., the mood-creativity correlations) that reflects the co-fluctuation of two or more variables over multiple recording times. Indeed, the matched sample results showed significant difference in several mood-creativity relationships by DRM and ESM data.

Here are several possible limitations of the present study. First, although self-report is possibly the most feasible approach in daily data collection and has been frequently applied in previous studies (Kahneman et al., 2004; Hofmann et al., 2014; Conner et al., 2018), a direction validation of the data quality is missing. Nevertheless, the consistency of our mood-creativity results with previous reports, provide indirect support for the validity of our data. Second, while the analysis of matched samples is interesting, caution must be taken for its interpretation: the matched DRM and ESM event could be different even if the time period overlapped, given the relative sparse event sampling in time. Third, the present results could not provide a causal explanation of the mood-creativity relationship, especially given the daily context: while mood states could predict state creativity, daily activities with different levels of creativity could also affect one’s mood states. Experimental controls would be needed to further elucidate this issue.

Nevertheless, the study introduced the DRM to the study of mood-creativity relationship for the first time. The DRM-based data showed a positive correlation between the positive mood states and creativity. The results were largely consistent with the simultaneously conducted ESM. This research suggests the effectiveness of using DRM for such a second-order statistical analysis. As DRM imposes a lessened respondent load on the participants as compared to ESM, our results suggest DRM as a promising tool for further mood-creativity research. Moreover, our results provide further empirical evidence toward a comprehensive understanding of mood and creativity.

## Data Availability

The datasets generated for this study are available on request to the corresponding author.

## Ethics Statement

This study was carried out in accordance with the recommendations of the Ethics Committee of the Department of Psychology, Tsinghua University with written informed consent from all subjects. All subjects gave written informed consent in accordance with the Declaration of Helsinki. The protocol was approved by the Ethics Committee of the Department of Psychology, Tsinghua University.

## Author Contributions

WH, KP, and DZ conceptualized and designed the study. WH, XF, and MZ conducted the experiment. XF and MZ performed the statistical analysis. WH and XF wrote the first draft of the manuscript. All authors wrote the sections of the manuscript and contributed to manuscript revision, read, and approved the final version of the manuscript.

## Conflict of Interest Statement

The authors declare that the research was conducted in the absence of any commercial or financial relationships that could be construed as a potential conflict of interest.

## References

[B1] Adaman J. E. Blaney P. H. (1995). The effects of musical mood induction on creativity. *J. Creat. Behav.* 29 95–108. 10.1002/j.2162-6057.1995.tb00739.x

[B2] Baas M. De Dreu C. K. Nijstad B. A. (2008). A meta-analysis of 25 years of mood-creativity research: hedonic tone, activation, or regulatory focus? *Psychol. Bull.* 134:779. 10.1037/a0012815 18954157

[B3] Bledow R. Rosing K. Frese M. (2013). A dynamic perspective on affect and creativity. *Acad. Manage. J.* 56 432–450. 10.5465/amj.2010.0894

[B4] Carlsson I. Wendt P. E. Risberg J. (2000). On the neurobiology of creativity. Differences in frontal activity between high and low creative subjects. *Neuropsychologia* 38 873–885. 10.1016/s0028-3932(99)00128-1 10689061

[B5] Clapham M. M. (2001). The effects of affect manipulation and information exposure on divergent thinking. *Creat. Res. J.* 13 335–350. 10.1207/s15326934crj1334_11

[B6] Conner T. S. DeYoung C. G. Silvia P. J. (2018). Everyday creative activity as a path to flourishing. *J. Posit. Psychol.* 13 181–189. 10.1080/17439760.2016.1257049

[B7] Conner T. S. Silvia P. J. (2015). Creative days: a daily diary study of emotion, personality, and everyday creativity. *Psychol. Aesthet. Creat. Arts* 9:463 10.1037/aca0000022

[B8] Davis M. A. (2009). Understanding the relationship between mood and creativity: a meta-analysis. *Organ. Behav. Hum. Decis. Proces.* 108 25–38. 10.1037/a0027652 22409506

[B9] De Dreu C. K. Baas M. Nijstad B. A. (2008). Hedonic tone and activation level in the mood-creativity link: toward a dual pathway to creativity model. *J. Pers. Soc. Psychol.* 94:739. 10.1037/0022-3514.94.5.739 18444736

[B10] Dockray S. Grant N. Stone A. A. Kahneman D. Wardle J. Steptoe A. (2010). A comparison of affect ratings obtained with ecological momentary assessment and the day reconstruction method. *Soc. Indicat. Res.* 99 269–283. 10.1007/s11205-010-9578-7 21113328 PMC2990978

[B11] George J. M. (2007). Creativity in organizations. *Acad. Manag. Ann.* 1 439–477.

[B12] George J. M. Zhou J. (2002). Understanding when bad moods foster creativity and good ones don’t: the role of context and clarity of feelings. *J. Appl. Psychol.* 87:687. 10.1037//0021-9010.87.4.687 12184573

[B13] George J. M. Zhou J. (2007). Dual tuning in a supportive context: joint contributions of positive mood, negative mood, and supervisory behaviors to employee creativity. *Acad. Manage. J.* 50 605–622. 10.5465/amj.2007.25525934

[B14] Göritz A. S. Moser K. (2003). Mood and flexibility in categorization: a conceptual replication. *Percept. Mot. Skills* 97 107–119. 10.2466/pms.97.5.107-119 14604029

[B15] Gough H. G. (1979). A creative personality scale for the adjective check list. *J. Pers. Soc. Psychol.* 37:1398 10.1037/0022-3514.37.8.1398

[B16] Hirt E. R. Levine G. M. McDonald H. E. Melton R. J. Martin L. L. (1997). The role of mood in quantitative and qualitative aspects of performance: single or multiple mechanisms? *J. Exp. Soc. Psychol.* 33 602–629. 10.1006/jesp.1997.1335

[B17] Hofmann W. Wisneski D. C. Brandt M. J. Skitka L. J. (2014). Morality in everyday life. *Science* 345 1340–1343. 10.1037/e512142015-287 25214626

[B18] Isen A. M. (1999). On the relationship between affect and creative problem solving. *Affect Creat. Exp. Psychol. Adjust.* 3 3–17.

[B19] Kahneman D. Krueger A. B. Schkade D. A. Schwarz N. Stone A. A. (2004). A survey method for characterizing daily life experience: the day reconstruction method. *Science* 306 1776–1780. 10.1126/science.1103572 15576620

[B20] Kaufman J. C. Sternberg R. J. (eds) (2010). *The Cambridge Handbook of Creativity.* Cambridge: Cambridge University Press.

[B21] Kim J. Kikuchi H. Yamamoto Y. (2013). Systematic comparison between ecological momentary assessment and day reconstruction method for fatigue and mood states in healthy adults. *Br. J. Health Psychol.* 18 155–167. 10.1111/bjhp.12000 23017062

[B22] Mikulincer M. Kedem P. Paz D. (1990). Anxiety and categorization—1. the structure and boundaries of mental categories. *Person. Individ. Differ.* 11 805–814. 10.1016/0191-8869(90)90189-x

[B23] Morrison D. M. Leigh B. C. Gillmore M. R. (1999). Daily data collection: a comparison of three methods. *J. Sex Res.* 36 76–81. 10.1080/00224499909551970

[B24] Muaremi A. Arnrich B. Tröster G. (2013). Towards measuring stress with smartphones and wearable devices during workday and sleep. *BioNanoScience* 3 172–183. 10.1007/s12668-013-0089-2 25530929 PMC4269214

[B25] Schwarz N. Clore G. L. (2003). Mood as information: 20 years later. *Psychol. Inquiry* 14 296–303.

[B26] Shalley C. E. Gilson L. L. (2004). What leaders need to know: a review of social and contextual factors that can foster or hinder creativity. *Leader. Q.* 15 33–53. 10.1016/j.leaqua.2003.12.004

[B27] Shiffman S. Stone A. A. Hufford M. R. (2008). Ecological momentary assessment. *Annu. Rev. Clin. Psychol.* 4 1–32. 10.1093/oxfordhb/9780199381708.013.1 18509902

[B28] Silvia P. J. Beaty R. E. Nusbaum E. C. Eddington K. M. Levin-Aspenson H. Kwapil T. R. (2014). Everyday creativity in daily life: an experience-sampling study of “little c” creativity. *Psychol. Aesthet. Creat. Arts* 8:183 10.1037/a0035722

[B29] Sternberg R. J. Lubart T. I. (1999). The concept of creativity: prospects and paradigms. *Handbook Creat.* 1 3–15. 10.1017/cbo9780511807916.003

[B30] Tierney P. Farmer S. M. (2002). Creative self-efficacy: its potential antecedents and relationship to creative performance. *Acad. Manag. J.* 45 1137–1148. 10.5465/3069429

[B31] To M. L. Fisher C. D. Ashkanasy N. M. Rowe P. A. (2012). Within-person relationships between mood and creativity. *J. Appl. Psychol.* 97:599. 10.1037/a0026097 22040262

[B32] Verhaeghen P. Joorman J. Khan R. (2005). Why we sing the blues: the relation between self-reflective rumination, mood, and creativity. *Emotion* 5:226. 10.1037/1528-3542.5.2.226 15982087

[B33] Vosburg S. K. (1998). The effects of positive and negative mood on divergent-thinking performance. *Creat. Res. J.* 11 165–172. 10.1207/s15326934crj1102_6

[B34] Zhou J. Shalley C. E. (2003). Research on employee creativity: a critical review and directions for future research. *Res. Pers. Hum. Res. Manag.* 22 165–217. 10.1016/s0742-7301(03)22004-1

